# Family characteristics associated with rural households’ willingness to renew the family doctor contract services: a cross-sectional study in Shandong, China

**DOI:** 10.1186/s12889-021-11048-5

**Published:** 2021-06-30

**Authors:** Wenjuan Li, Jie Li, Peipei Fu, Yan Chen, Yemin Yuan, Shijun Yang, Jie Li, Zhixian Li, Chen Yan, Zhen Gui, Chengchao Zhou

**Affiliations:** 1 Centre for Health Management and Policy Research, School of Public Health, Cheeloo College of Medicine, Shandong University, Jinan, 250012 China; 2 School of Public Health, Wannan Medical College, Wuhu, 241002 China; 3 Department of Epidemiology, School of Public Health, Cheeloo College of Medicine, Shandong University, Jinan, 250012 China; 4 NHC Key Laboratory of Health Economics and Policy Research, Shandong University, Jinan, 250012 China

**Keywords:** Family characteristics, Renewal willingness, Family doctor contract services, Cross-sectional study

## Abstract

**Background:**

In China, some previous studies have investigated the signing rate and willingness of residents to sign the family doctor contract services (FDCS). Few studies have explored residents’ willingness to renew the FDCS. This study is designed to understand the family characteristics difference towards rural households’ willingness of maintaining the FDCS.

**Methods:**

A total of 823 rural households were included in the analysis. A descriptive analysis was conducted to describe the sample characteristics. The binary logistic regression model was used to explore the family characteristics that influence the renewal willingness for FDCS among rural households in Shandong province, China.

**Results:**

Our study found that about 95.5% rural households had willingness to maintain the FDCS in Shandong, China. Those households with catastrophic health expenditures (CHE) (OR = 0.328, 95%CI = 0.153–0.703), with highest level of education at graduate or above (OR = 0.303, 95%CI = 0.123–0.747) were less willing to maintain the FDCS. Those whose households have more than half of the labor force (OR = 0.403, 95%CI = 0.173–0.941) and those households living in economically higher condition were less willing to maintain the FDCS.

**Conclusions:**

This study demonstrates a significant association between family characteristics (CHE, highest education in households, proportion of the household labor force) and willingness to maintain FDCS among rural households in Shandong, China. Targeted policies should be made for rural residents of identified at-risk families.

**Supplementary Information:**

The online version contains supplementary material available at 10.1186/s12889-021-11048-5.

## Introduction

The declaration of Alma Ata in 1978, issued by the WHO, emphasized the significant of primary healthcare (PHC) in strengthening the health systems of all countries [1]. In 2009, China launched a new round of health system reform, in which strengthening the construction of the PHC system was one of key measures [2]. In 2011, the State Council issued the Guiding Opinions on Establishing a General Practitioner System [3], which proposed the establishment of a general practitioner system and a hierarchical medical system, and the implementation of general practitioner services. Family doctor contract services (FDCS) aims to provide comprehensive health management services. The FDCS is provided by a family doctor service team composed of general practitioners as the core members. From the perspective of residents’ health needs, family doctors provide comprehensive and whole-process health services and manage the health of residents within their jurisdiction by means of contracts, in accordance with the principles of informed, voluntary and family units and free choice [4]. In May 2016, the State Council’s Medical Reform Office, the National Health Commission, the National Development and Reform Commission, and seven other departments jointly issued the Guiding Opinions on Promoting Family Doctor Contract Services [5], China has begun to promote contracts with general practitioner services on a large scale.

In order to implement the Guidance Opinions on Promoting Family Doctor Contract Services, Shandong province issued the Implementation Advice on Accelerating and Improving Family Doctor Contract Services in December 2016, marking that Shandong province began to comprehensively promote the signing service system of family doctors [6]. It was proposed in the Guidance that all counties (cities and districts) in the province should promote and improve the pilot work of FDCS. In addition, the coverage of FDCS in the next year should reach 30% in urban areas and 65% in rural areas, the service coverage rate of key groups reached over 60% [6]. Signing a contract with the family doctor is voluntary, the contract deadline for 1 year after the expiry of the contract, residents can renew the contract with their family doctors, also can choose to sign with another family doctor, or terminate the contract [7]. Even though there are mandatory family doctor contracts in European and American countries, there are usually several medical centers for residents to choose from and sign up for [8, 9]. In this case, the number of residents who sign a contract with a family doctor is directly related to the income and career development of the family doctor. To some extent, this can objectively reflect the ability and level of diagnosis and treatment of family doctors in PHC [10].

Family physician plans are one of the most effective methods to improve people’s fair access to health services and are considered one of the major reforms of health system development [11]. The research on family doctors in China mainly focused on the significance of establishing the family doctor system and the difficulties in its implementation [12], or the evaluation of implementation effect of the family doctor policy. Assessment of the effectiveness of the implementation of the family doctor policy, which mainly includes assessment of the effectiveness of the health aspects such as the management of the residents’ chronic diseases [13–15], residents’ cognition of FDCS [16], residents’ demands for FDCS [17], to the family doctor service satisfaction as well as residents of the family doctor service quality evaluation and trust [18, 19]. At the same time, some scholars have focused their attention on the signing rate and willingness of residents to sign the FDCS in China.

At present, the form of signing FDCS take the family as the units in Shandong province. It indicates that family characteristics are an important research perspective. However, there are few studies on residents’ willingness to renew the FDCS, and the only few studies on this aspect mainly focus on the interviewees’ personal characteristics [20–23], while few studies on residents’ family characteristics are carried out. According to the planning requirements, by 2020, we will strive to expand the contracted services to the entire population, form a long-term and stable contractual service relationship, and basically realize the full coverage of the family doctor’s contracted service system [5]. The signing agreement is the bond between the general practitioner and the residents, which can consolidate the contracted service relationship between the residents and the family doctor service team [4]. Therefore, it is of great practical significance how to ensure the renewal of the contract of the contracted patients while steadily improving the contract rate, so as to ensure that the improvement of the overall signing rate and the implementation of the family signing system [24]. In the current research, the signing rate is mostly used as the main evaluation indicator to measure the contract signing of residents. However, compared with the signing rate, the subjectivity and prospect of the contracted residents’ willingness to renew the family doctor can make them more capable of evaluating the contracted residents [25]. This study aims to explore the influencing factors of the willingness to renew the family doctor contract services among rural households in Shandong, China. To do so, we have several specific objectives. First, to know the rate of rural households renewing their FDCS among those who have already signed the contract. Second, to explore the factors influencing the rural families to renew the contract of family doctor service from the perspective of the family characteristics.

## Methods

### Data source and sample

This study was conducted in Shandong, China. Shandong ranks the second in the population size in China. It has coastal and inland areas, and presents differences in economic development in different areas, which is similar and also a miniature of the whole China. A family-based cross-sectional study was conducted in Shandong province, China in 2018. Firstly, three cities (Zibo, Binzhou, and Liaocheng) were randomly selected according to the high, medium and low levels of economic development in Shandong province through multistage stratified cluster sampling. Secondly, 2 counties (districts) were randomly selected from each city, and 5 townships were randomly selected from each county, then 5 villages were randomly selected from each township, and 16 households were randomly selected from each village for this study by using random numbers. Therefore, we selected 180 villages in six counties and three cities in total. The researchers conducted a questionnaire survey after the acquisition of the informed consent of the respondents. If there were two or more eligible rural residents from one family, only one was randomly selected to participate in the survey (generally the head of a household). A total of 2979 questionnaires were collected.

**Fig. 1:**
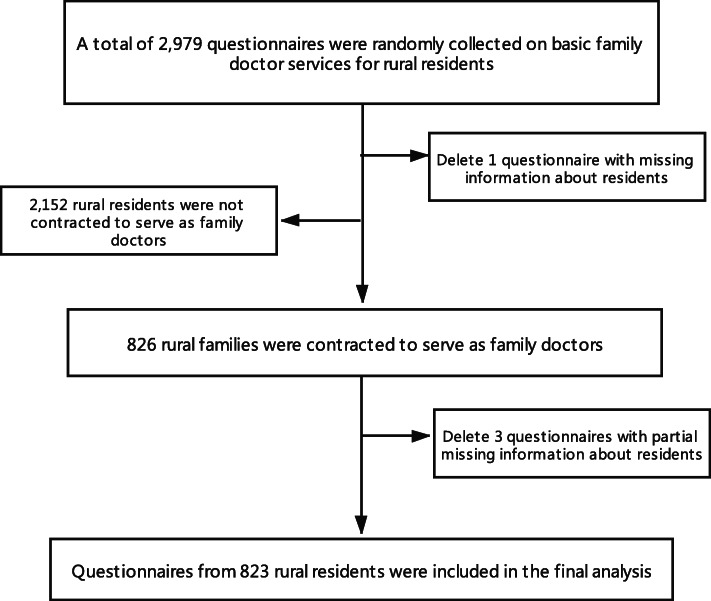
The flowchart for the sampling in this study

The participants in this study were filtered by one question: “Did you contract with the family doctors this year?” Those samples with the answer “Yes” were included in this study. Among the 2979 rural households, 826 households who had signed up for the FDCS were the subjects of this study. In addition, 3 questionnaires were discarded due to the absence of some key variables. Finally, 823 qualified questionnaires were included in the analysis (see Fig. 1 for details). All participants accepted face-to-face interviews by trained postgraduate students from the school of public health, Shandong University. All interviewers received adequate training to ensure the reliability of the survey. The questionnaire included basic family information, quality of life and chronic diseases, health behavior, health services use, access to health services, and family doctor contract and demand. Written informed consents were obtained from all of the participants before they participated in this study. This study protocol was approved by the ethics committee of School of Public Health, Shandong University, China.

The sample size calculation was performed by the following formula: $\mathrm{n}=\frac{{\mathrm{u}}_{\upalpha}^2\uppi \left(1-\uppi \right)}{\updelta^2}\ \left(\uppi :\mathrm{expected}\ \mathrm{prevalence}\right)$ [26]. The contracted rate was *p* = 47% according to previous studies [27], so in the study of the current situation of residents signing contracts with family doctors, π = 0.47, μα = 1.96, δ = 0.02, α = 0.05. We found that the required sample size was 2392. The total sample size obtained in our study was 2979, which has reached the required sample size. This study aims to explore the willingness of contracted residents to renew their family doctors. According to previous studies, contract renewal rate π = 93.4% [28], μα = 1.96, δ = 0.02, α = 0.05, we found that required sample size was 529. Therefore, our sample is representative and sufficient.

### Measurement

In this study, in order to ensure the authenticity and validity of the survey data, quality control runs through the entire process of on-site investigation, including design and pilot study, investigator training, on-site investigation, data review, data entry, data management and other aspects of the investigation work. In addition, we also validated the self-reported FDCS signing status of the participants with the family doctors in the sampling villages.

#### Dependent variable

The dependent variable of this study was measured by asking the respondents “Are you willing to renew your contract in next year?” The answer was “Yes” or “No”.

#### Independent variables

The independent variables in this study were a series of variables that described the characteristics of rural families, the selection of family characteristic variables was based on a literature review and expert consultation, including:(1) city of residence (Zibo, Binzhou, and Liaocheng); (2) poor family, which was measured by a question of “Is your family a poor household?” The answer was “Yes” or “No”; (3) family income, which refers to the annual household income of the sample households in 2017, divided into ≤30,000 yuan or > 30,000 yuan, the median of the household income of the participating families; (4) catastrophic health expenditure (CHE) [29], which was defined as whether the proportion of a household’s medical expenditure to total household expenditure (excluding food expenditure) exceeds 0.4 (including 0.4); (5) the time to the nearest village clinic [30], which was measured by a question: “How long does it take from your home to nearest village clinic (the most common mode of transportation)?” According to this question, we divided the time into≤10 min or > 10 min; (6) highest education in household, which refers to the highest level of education among family members. The educational level was divided into junior high school or below, senior high school, and graduate or above [31, 32]; (7) proportion of the household labor force, which was classified according to whether the household labor force accounts for more than half of the total household size [33, 34]. The household labor force population was defined as the family members aged between 18 and 65; (8) pay for the family doctor service contract, which was measured by a question of “How much do you pay for the FDCS (based on the family unit) every year?” We divided the variable into the binary variable based on whether the respondents pay or not. (9) the number of members having chronic diseases in household; (10) pregnant woman in household, which was measured by a question of “Have you had a pregnant woman in your family for the past two years?” (11) children in household, which was measured by a question of “Do you have children aged 0-6 years in your family?” (12) persons aged 65 years or over in household, which was measured by a question of “Are there any elderly adults 65 or older in your family?”

### Statistical analysis

A descriptive analysis was conducted to describe the sample characteristics. Mean and standard deviation were calculated for continuous variables, and frequencies and percentages for categorical variables. A comparison of the family characteristics between the rural households who were willing to renew FDCS and those who were unwilling to was conducted using the Chi-square (χ2) test. The significance level was set as *P* < 0.05 (two-sided). We incorporated family characteristics with *P* < 0.1 in the results of χ2 tests into the logistic regression model. We used a stepwise selection method to select variables that were associated with rural households’ willingness to renew their FDCS (the criterion for selecting and eliminating variables were *P* < 0.05 and *P* < 0.10, respectively). Through the above methods, we explore the independent associations between family factors with the rural households’ willingness to maintain the family contracts, and the threshold of statistical significance was set at *P* < 0.05 (two-tailed). The odds ratio (OR) and 95% confidence interval (CI) of the variables were reported. In this study, EpiData version 3.1 software (EpiData Association, Odense, Denmark, Europe) was used to establish the database, and the double para entry rule was applied for inputting data. Before data entry, we provided a unified entry manual for each entry clerk, and conducted necessary training. In order to ensure the quality, two entry clerks double record and compare. After data entry being completed, spot check is carried out. SPSS 26.0 statistical analysis software and Stata 16.0 (Stata Corp, College Station, TX, USA) were used to conduct the analyses.

## Results

### Sample characteristics

Among the 823 interviewed rural households (Table 1), 41.8% (344) were located in Zibo, 18.3% (151) were located in Binzhou, and 39.9% (328) were located in Liaocheng. Nearly 8.8% (72) rural households were poor, 46.4% (382) of households had annual incomes above 30,000 yuan, and 34.1% (281) of rural households have incurred CHE. More than 90% of the families spent less than 10 min to reach the village clinic (including 10 min). As for highest education in households, 57.6% (474) were junior high school or below, 27.5% (226) were senior high school and only 14.9% (123) were graduate or above. The proportion of the household labor force beingless than 0.5 (including 0.5) accounted for 47.8%. Among rural families that had signed up family doctors, 97% of families didn’t pay for the FDCS. In term of rural family members suffering from chronic diseases, 38.6% (318) of families had no members with chronic diseases, and only 1.3% (11) of families had three or more members with chronic diseases. Additionally, 11.7% (96) had pregnant woman, 21.5% (177) had children and 46.8% (385) had 65 years old or older adults in the family.

**Table 1:** Sample characteristics by residents’ willingness to maintain contracts with family doctors

		Renewal willingness			
Variables	Total n (%) (*n* = 823,100%)	Willing n (%) (*n* = 786,95.5%)	Unwilling n (%) (*n* = 37,4.5%)	χ2	*P-*value
City of residence				19.331	< 0.001
Zibo	344 (41.8)	316 (91.9)	28 (8.1)		
Binzhou	151 (18.3)	146 (96.7)	5 (3.3)		
Liaocheng	328 (39.9)	324 (98.8)	4 (1.2)		
Poor family				–	0.556
No	751 (91.3)	718 (95.6)	33 (4.4)		
Yes	72 (8.7)	68 (94.4)	4 (5.6)		
Family income (yuan)				3.862	0.049
≤ 30,000	441 (53.6)	427 (96.8)	14 (3.2)		
> 30,000	382 (46.4)	359 (94.0)	23 (6.0)		
Catastrophic health expenditure				3.625	0.057
No	542 (65.9)	523 (96.5)	19 (3.5)		
Yes	281 (34.1)	263 (93.6)	18 (6.4)		
The time to the nearest village clinic (minutes)				–	0.399
≤ 10	742 (90.2)	710 (95.7)	32 (4.3)		
> 10	81 (9.8)	76 (93.8)	5 (6.2)		
Education, highest in household				12.662	0.002
Junior high school or below	474 (57.6)	459 (96.8)	15 (3.2)		
Senior high school	226 (27.5)	217 (96.0)	9 (4.0)		
Graduate or above	123 (14.9)	110 (89.4)	13 (10.6)		
Proportion of the household labor force				6.670	0.010
≤ 0.5	393 (47.8)	383 (97.5)	10 (2.5)		
> 0.5	430 (52.2)	403 (93.7)	27 (6.3)		
Pay for the family doctor service contract				–	0.022
No	798 (97.0)	765 (95.9)	33 (4.1)		
Yes	25 (3.0)	21 (84.0)	4 (16.0)		
The number of chronic diseases in household				1.355	0.454
0	318 (38.6)	305 (95.9)	13 (4.1)		
1–2	494 (60.0)	471 (95.3)	23 (4.7)		
≥ 3	11 (1.3)	10 (90.9)	1 (9.1)		
Pregnant woman in household				–	0.112
No	727 (88.3)	691 (95.0)	36 (5.0)		
Yes	96 (11.7)	95 (99.0)	1 (0.1)		
Children in household				4.120	0.042
No	646 (78.5)	612 (94.7)	34 (5.3)		
Yes	177 (21.5)	174 (98.3)	3 (1.7)		
Persons aged 65 or over in household				0.011	0.917
No	438 (53.2)	418 (95.4)	20 (4.6)		
Yes	385 (46.8)	368 (95.6)	17 (4.4)		

**Note: "-"** Use Fisher’s exact probability method, there is no corresponding statistical value

### Factors associated with rural households’ willingness to maintain contracts with family doctors

Results of the univariate analysis suggested the city of residence, household income, highest level of education in households, proportion of the household labor force, whether paid for the family doctor service contract, and whether the family had children aged 0–6 years old were significantly related to rural households’ renewal willingness (*P* < 0.05) (Table 1).

The results of the binary stepwise logistic regression analysis on the influencing factors of rural households’ willingness to maintain the FDCS summarized in Table 2. In order to better explore the factors associated with the willingness of rural households to maintain the FDCS, we incorporated variables describing family characteristics into the model with *P* < 0.1 as the result of univariate analysis.

**Table 2:** Binary stepwise logistic regression analysis on the influencing factors of rural households’ willingness to maintain contracts with family doctors

Variables	Model		
	Odds Ratio	*P*-value	95%CI
City of residence			
Zibo(ref.)	1		
Binzhou	1.900	0.209	(0.698,5.169)
Liaocheng	5.897	0.001	(2.020,17.217)
Catastrophic health expenditure			
No(ref.)	1		
Yes	0.328	0.004	(0.153,0.703)
Education, highest in household			
Junior High school or below(ref.)	1		
Senior high school	0.779	0.594	(0.311,1.951)
Graduate or above	0.303	0.010	(0.123,0.747)
Proportion of the household labor force			
≤ 0.5(ref.)	1		
> 0.5	0.403	0.036	(0.173,0.941)
Children in household			
No(ref.)	1		
Yes	3.410	0.052	(0.991,11.739)

*Ref* reference group

Results showed that family characteristics, including city of residence, CHE, highest education in households, and proportion of the household labor force, were statistically associated with the rural households’ willingness to maintain the FDCS. In detail (Table 2), compared with the households living in Zibo, those households living in Liaocheng, where the economy is poorer, were more willing to maintain the FDCS (OR = 5.897, 95%CI = 2.020–17.217). Those households with CHE were less willing to maintain the FDCS than those without CHE (OR = 0.328, 95%CI = 0.153–0.703). Those households with highest level of education at graduate or above were less willing to maintain the FDCS than those with the highest level of education at junior high school or below (OR = 0.303, 95%CI = 0.123–0.747). Those whose households have more than half of the labor force were less willing to maintain the FDCS (OR = 0.403, 95%CI = 0.173–0.941).

### Robustness checks

In the robustness test, we have compared the parameter estimation between the general logistic regression model and the rare logistic regression model. We find that the results of parameter estimation do not change significantly. (See the Supplementary file 1).

## Discussion

In the current study, approximate 95.5% of rural households who contracted with family doctors were willing to maintain the FDCS, which is higher than the results in a national survey in 2019 (71.3%) [22]. This rate is also higher than those of the urban residents in Zhejiang province in 2019 (91%) [21] and in Yichang of Hubei province in 2017 (85.9%) [20], and higher than those of rural residents in Lanzhou in 2018 (82%) [23]. This indicates that the implementation of the policy for rural residents’ family doctors in Shandong province might have achieved relatively good results, and the residents have higher utilization experience in terms of the quality of family doctors’ health care services [22]. At the same time, it is worth noting that even though the households who have signed up with FDCS are up to 96% willing to renew their contract, the current signing rate for FDCS among the rural residents was rather low (27.7%). This result indicates that after signing up for FDCS, residents may increase the rate of signing up for FDCS, because they have experienced related family doctor services or have a better knowing of FDCS [35, 36]. Therefore, it suggests that in pushing forward the FDCS policy in rural Shandong in the future, the government should pay more attention to the implementation of the propaganda work, so that rural residents can learn more about the family doctor policy. At the same time, the policy should provide more opportunities for contracted residents to enjoy the FDCS, which might be one of the effective ways to improve the rate of residents signing the FDCS.

Family without CHE are more willing to maintain the FDCS. On the one hand, the increase in the number of people living with chronic diseases and hospitalizations in the family are risk factors for CHE [37]. Hospital care is still the preferred choice for the majority of residents with chronic diseases, possibly because they trust the quality of medical care in higher level hospitals more. Those households are still reluctant to make use of FDCS, even if they have signed up, they still consider PHC ineffective and believe that PHC doctors do not have enough skills to treat their diseases [38] On the other hand, since rural families who have signed FDCS are our research objects, if those families have CHE, it also may lead to a decrease in the residents’ trust in FDCS and believe that FDCS are not helpful to their families, and satisfaction with FDCS decreased [39].

The highest level of family education is negatively correlated with the willingness of rural households to maintain FDCS. This finding is consistent with previous studies, which indicated that residents with an education level of junior high school or below were more willing to sign up for FDCS than those of other education groups [4, 40, 41]. Highly educated residents pay more attention to their own health and are more inclined to go to higher level hospitals for medical treatment due to the lack of trust in PHC [42]. Our study shows that there was a significant difference in renewing FDCS between rural households with highest education level of junior high school or below and those with college or above, but not statistically significant difference with high school. This may be related to the relationship between the most educated members in the family. The members of rural family in China are mostly educated at the primary school level or below. Those with a high school or university degree level or above are generally offspring of the families. According to China’s education system, the educated family members at the university level or above are almost all in the state of adults, and they have a large influence on the decision-making in the family.

Compared with rural households with a household labor force ratio of more than 0.5, we find that rural households with a household labor force ratio of 0.5 or below are more willing to maintain the FDCS. We know that the family members above 65 or below the age of 18 may make greater use of the FDCS as these two groups may possibly have greater demand for health services [42]. Meanwhile, the rural families with children aged 0–6 years old were more willing to renew contracts with family doctors, which is consistent with our hypothesis, although this is not a significantly independent factor in our model. As a result, they will perhaps experience more relevant policy services, which is the reason for the higher maintenance of contracted services. In addition, rural families with a labor force ratio above 0.5 generally have a better economic level and may prefer to go to a large hospital for treatment.

The findings in the current study imply that we should pay more attention to those families with CHE, highly educated and a large labor force, because these families have a lower willingness to maintain the FDCS. Therefore, the government should strengthen the publicity of FDCS and enhance the residents’ awareness of significance of FDCS use, so as to improve the recognition and trust of FDCS. Next, the government should further implement the policies to support the FDCS, including expanding service packages, providing more personalized FDCS, so as to further promote the sustainable development of the contract service system of family doctors.

### Limitations

This study has some limitations. Firstly, the cross-sectional design of this study could only interpret the association of rural households’ willingness to maintain the FDCS and family characteristics, limiting its ability to identify the causal relationships between influencing factors and residents’ willingness. Secondly, some other factors that might impact households’ willingness were not included in our survey, such as family structure variables, which needs to be remedied in the future study. Thirdly, this study was conducted in Shandong province, and there is a lack of other regional or national investigations on this aspect, so the representativeness is insufficient.

## Conclusion

This study finds that there is a significant correlation between the characteristics of rural families in Shandong province and the intention to maintain the FDCS. Sufficient attention should be paid to the lower level of willingness to renew family doctor contracts of rural households with CHE, more than half of the household labor force ratio, the higher level of family education. The government should provide more policy support, increase publicity, expand service packages, so as to further promote the sustainable development of the contract service system of family doctors.

## Supplementary Information

**Additional file 1: Table 1.** General logistic regression parameter estimation. **Table 2.** Logistic regression parameter estimation for rare event correction

## Data Availability

The datasets used in the current study are available from the corresponding author (Prof. Chengchao Zhou) on reasonable request.

## Abbreviations

FDCS: Family doctor contract services
PHC: Primary healthcare
CHE: Catastrophic health expenditure
