# Supplementary material for: Family characteristics associated with rural households’ willingness to renew the family doctor contract services: a cross-sectional study in Shandong, China
Source: BMC Public Health. 2021 Jun 30;21:1282. doi: 10.1186/s12889-021-11048-5 (PMC8246675; doi:10.1186/s12889-021-11048-5)
Supplement: Supplementary file 1 — Additional file 1: Table 1. General logistic regression parameter estimation. Table 2. Logistic regression parameter estimation for rare event correction [file 12889_2021_11048_MOESM1_ESM.docx]

**Supplementary file 1**

Table 1 General logistic regression parameter estimation

|  | β | S_b_ | Z | P | OR |
| --- | --- | --- | --- | --- | --- |
| intercept | 4.334 | 0.956 | 4.54 | P<0.001 |  |
| City of residence | 0.852 | 0.249 | 3.42 | 0.001 | 2.343 |
| Catastrophic health expenditure | -1.108 | 0.386 | -2.87 | 0.004 | 0.330 |
| Education, highest in household | -0.586 | 0.237 | -2.48 | 0.013 | 0.556 |
| Proportion of the household labor force | -0.845 | 0.426 | -1.99 | 0.047 | 0.429 |
| Children in household | 1.246 | 0.628 | 1.99 | 0.047 | 3.477 |

Table2 Logistic regression parameter estimation for rare event correction

|  | β | S_b_ | Z | P | OR |
| --- | --- | --- | --- | --- | --- |
| intercept | 4.247 | 0.984 | 4.32 | P<0.001 |  |
| City of residence | 0.814 | 0.242 | 3.36 | 0.001 | 2.257 |
| Catastrophic health expenditure | -1.091 | 0.376 | -2.90 | 0.004 | 0.336 |
| Education, highest in household | -0.579 | 0.242 | -2.39 | 0.017 | 0.560 |
| Proportion of the household labor force | -0.813 | 0.383 | -2.12 | 0.034 | 0.444 |
| Children in household | 1.085 | 0.631 | 1.72 | 0.086 | 2.959 |

**Reference:**

Zhao J, Luo T, Fan Y, Zeng P, Chou L, Liu G: The Application of Rare Event Logistic Regression in Medical Research. Chinese Journal of Health Statistics 2011, 28(06):641-644.

Gao Y, Zhang J: Determination of Sample Size in Logistic Regression Analysis. The Journal of Evidence-Based Medicine 2018, 18(02):122-124.

Tomz M, King G, Zeng L: ReLogit: Rare Events Logistic Regression. Journal of Statistical Software 2003, 008.
